# Supplementary material for: Reduced γ-glutamyl hydrolase activity likely contributes to high folate levels in Periyakulam-1 tomato
Source: Hortic Res. 2022 Nov 19;10(1):uhac235. doi: 10.1093/hr/uhac235 (PMC9832877; doi:10.1093/hr/uhac235)
Supplement: Web_Material_uhac235 [file web_material_uhac235.zip › Figure S1-S8.pdf]

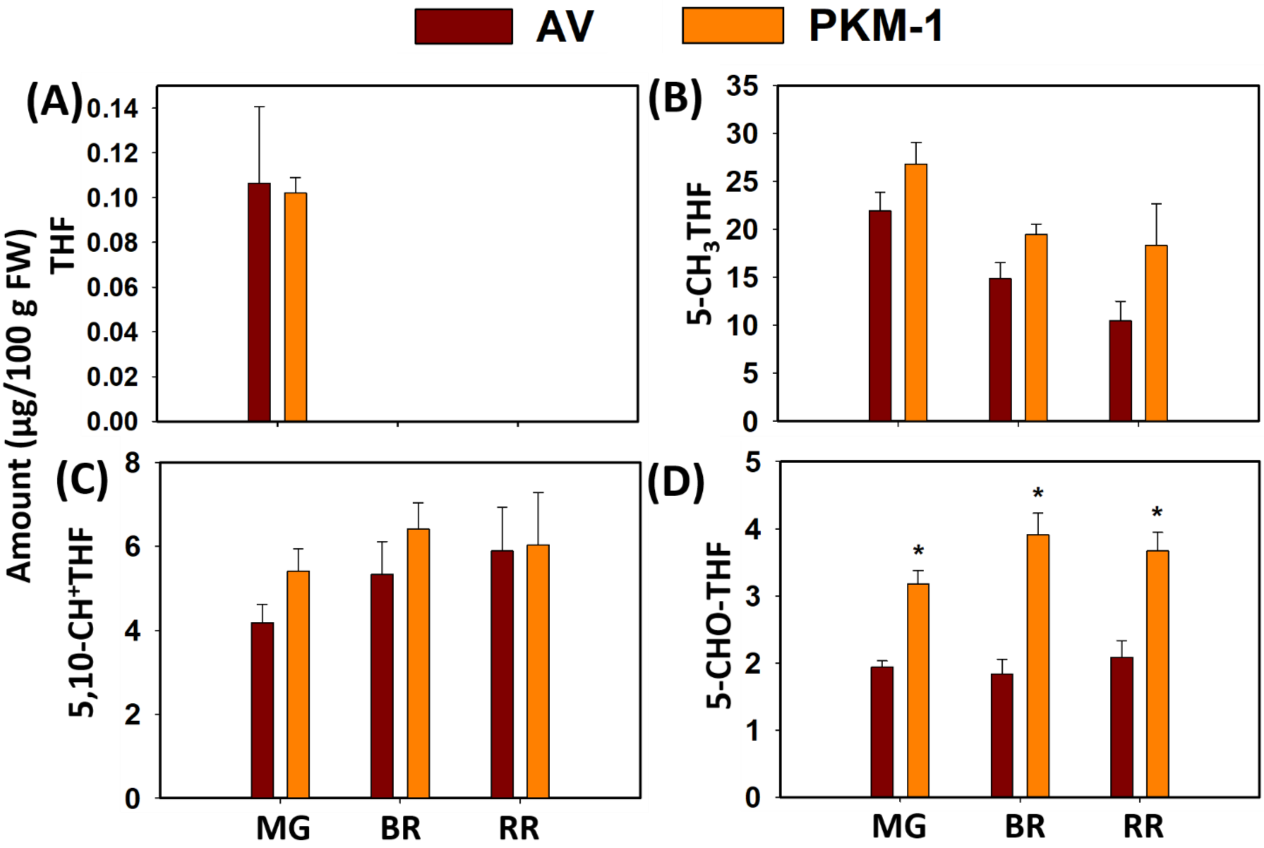


**Figure S1. Folate profiling in AV and PKM-1 during ripening. (A)** THF, **(B)** 5-$CH_3$ THF, **(C)** 5,10-$CH^+$ THF, and (D) 5-CHO-THF at different ripening stages. Data are means ± SE ($n \geq 3$ ), * = $P \leq 0.05$. An asterisk (*) shows a significant difference compared to AV. Abbreviations : THF, tetrahydrofolate; 5-$CH_3$ THF, 5-methyltetrahydrofolate; 5,10-$CH^+$ THF, 5,10-methenyltetrahydrofolate; 5-CHO-THF, 5-Formyltetrahydrofolate .

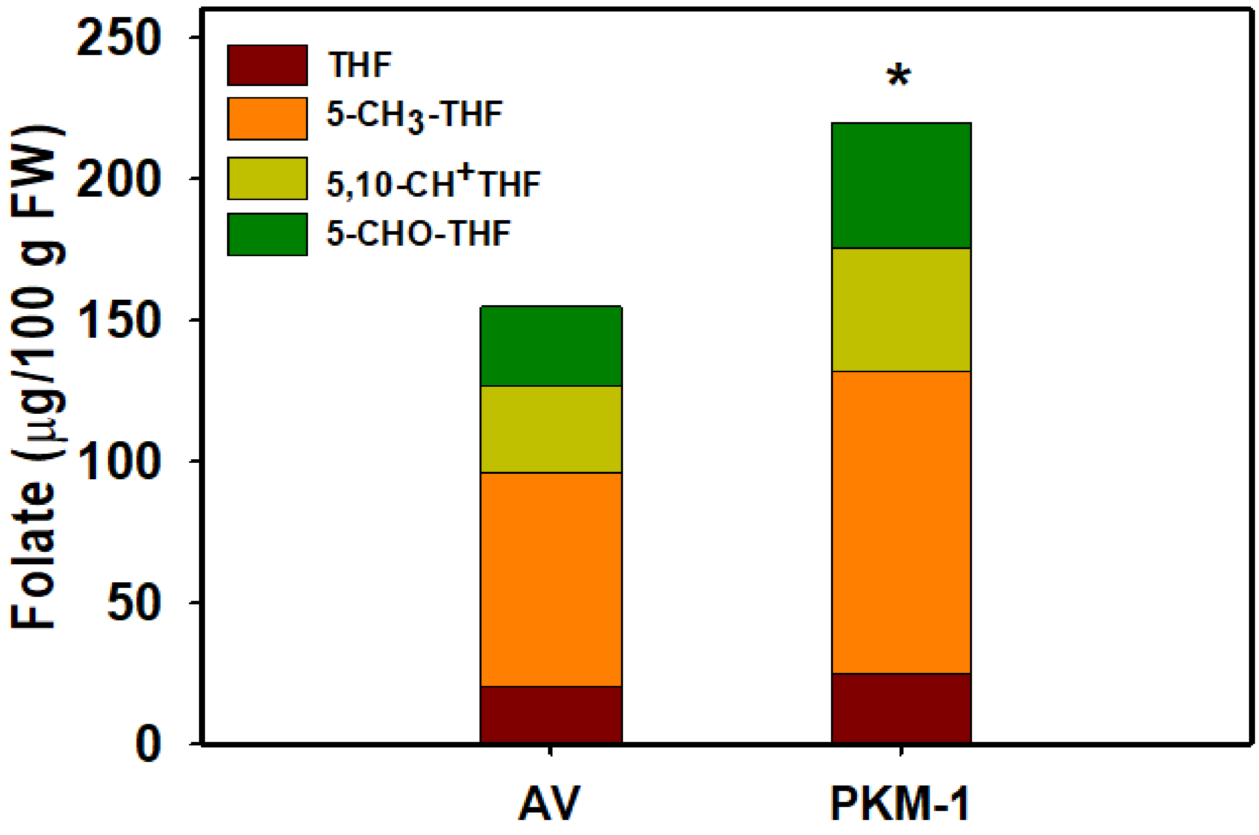


**Figure S2. Total folate levels in AV and PKM-1 leaves.** The leaves harvested from the $5^{th}$ node of 7-week-old AV and PKM-1 plants were used for folate estimation using LC-MS. Data are means ± SE (n = 5), * = $P \leq 0.05$. An asterisk (*) shows a significant difference compared to AV Abbreviations: THF, tetrahydrofolate; 5-$CH_3$THF, 5-methyltetrahydrofolate; 5,10-$CH^+$THF, 5,10-methenyltetrahydrofolate; 5-CHO-THF, 5-Formyltetrahydrofolate.

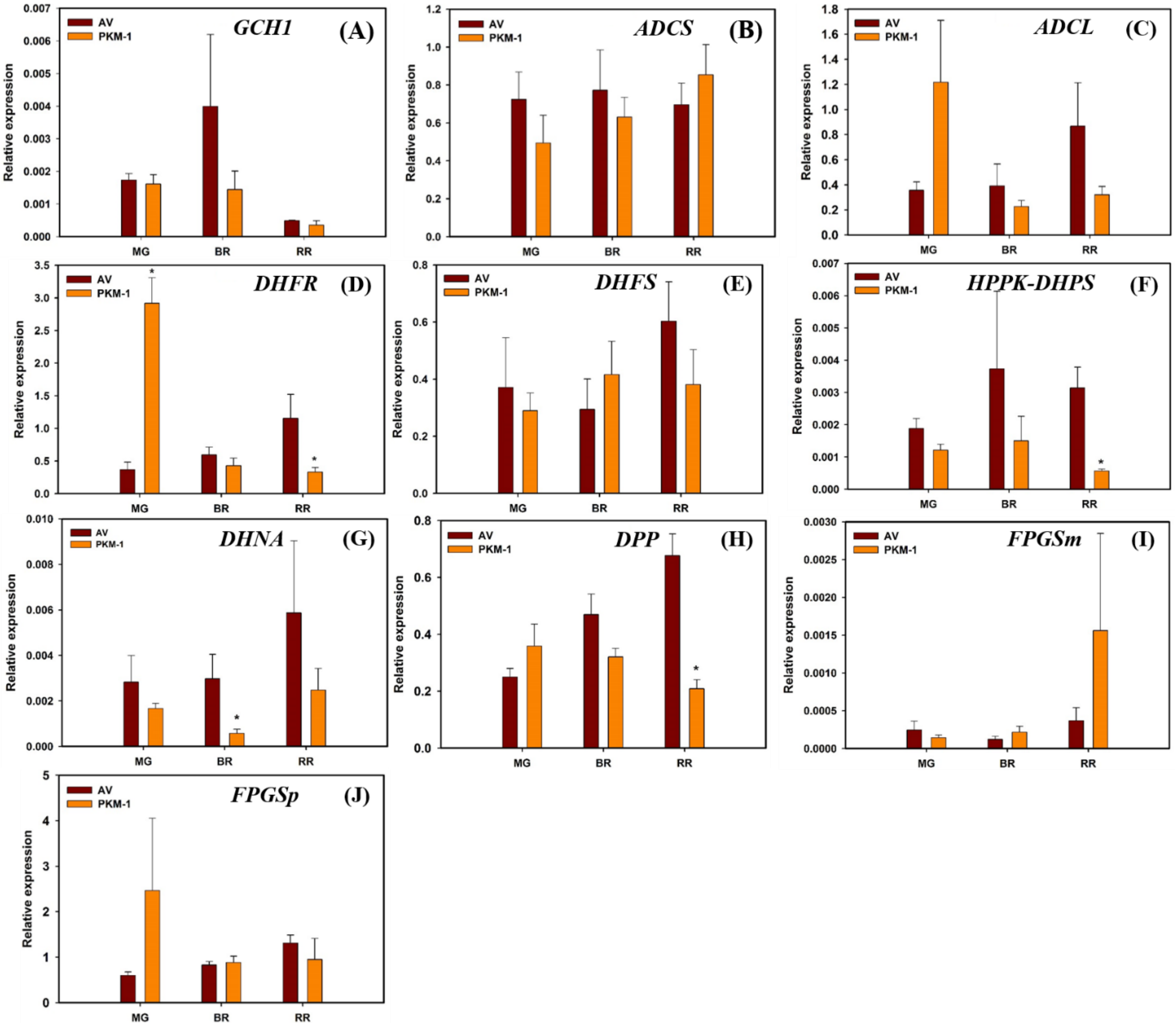


**Figure S3. The expression of folate biosynthesis pathway genes in AV and PKM-1 fruits.** Data are means ± SE (n = 3), * = P ≤ 0.05 . An asterisk (*) shows a significant difference compared to AV Abbreviations: *GCHI*, GTP cyclohydrolase I; *DPP*, dihydroneopterin (DHN) triphosphate diphosphatase; *DHNA*, DHN aldolase; *ADCS*, aminodeoxychorismate (ADC) synthase; *ADCL*, aminodeoxychorismate lyase; *HPPK-DHPS*, 6-hydroxymethyl-7,8 dihydropterin (HMDHP) pyrophosphokinase (HPPK) and dihydropteroate (DHP) synthase; *DHFS*, dihydrofolate (DHF) synthase; *DHFR*, DHF reductase; *FPGS*, folylpolyglutamate synthase (p-plastic, m-mitochondrial).

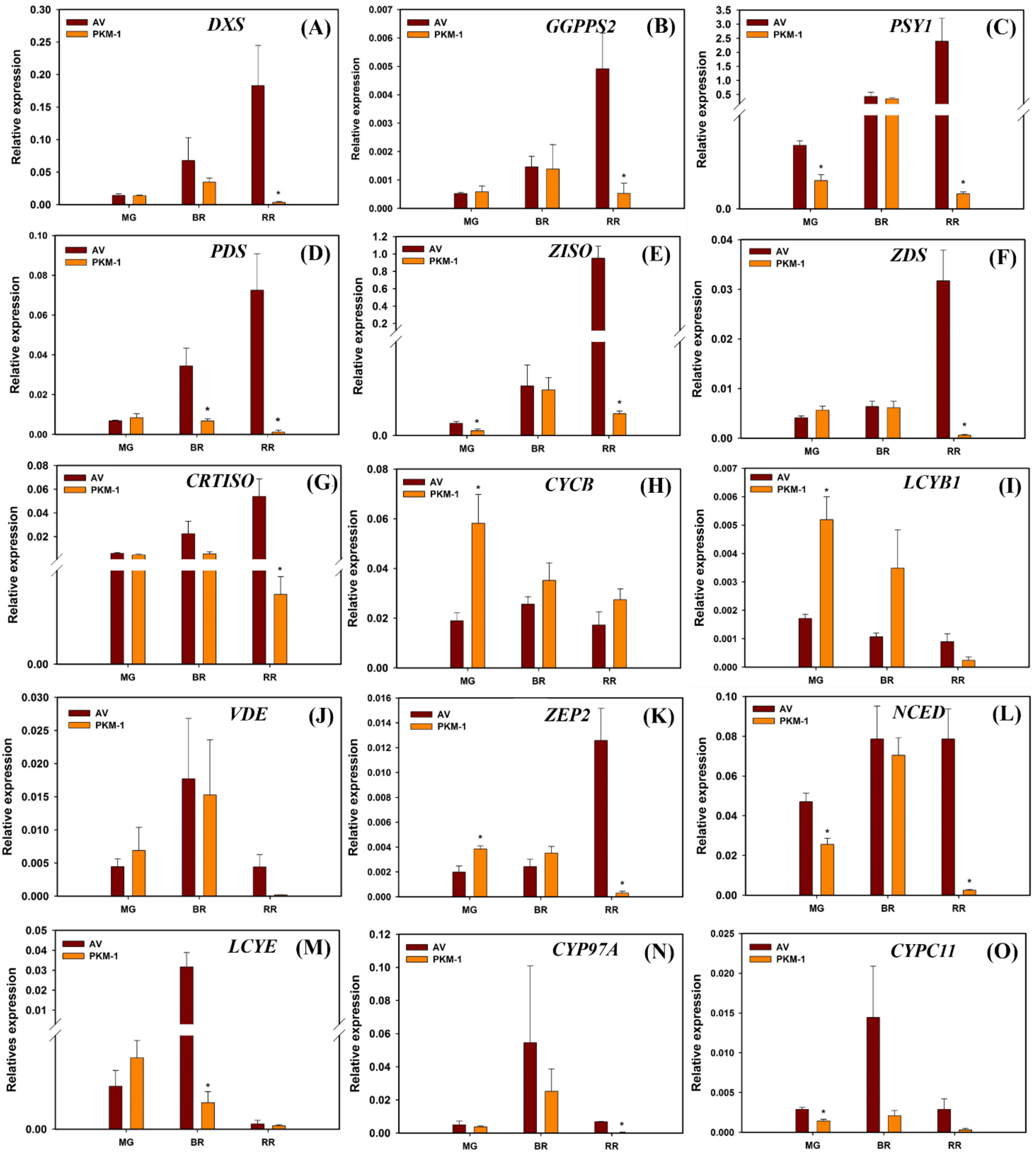


**Figure S4. Carotenoid biosynthesis pathway gene expression in AV and PKM-1 fruit during ripening.** Data are means ± SE (n = 3), * = P ≤ 0.05. Abbreviations: *DXS*, 1-deoxy-d-xylulose-5-phosphate synthase; *GGPPS2*, geranylgeranyl diphosphate synthase; *PSY1*, phytoene synthase; *PDS*, phytoene desaturase; *ZISO*, ζ-carotene isomerase; *ZDS*, ζ-carotene desaturase; *CRTISO*, carotenoid isomerase; *CYCB*, lycopene β-cyclase (chromoplastic); *LCYB1*, lycopene β-cyclase; *VDE*, violaxanthin de-epoxidase, *ZEP*, zeaxanthin epoxidase; *NCED*, 9-cis-epoxycarotenoid dioxygenase; *LCYE*, lycopene ε-cyclase, *CYP97A*, cytochrome P450 carotenoid β-hydroxylase; *CYPC11*, cytochrome P450 carotenoid ε-hydroxylase.

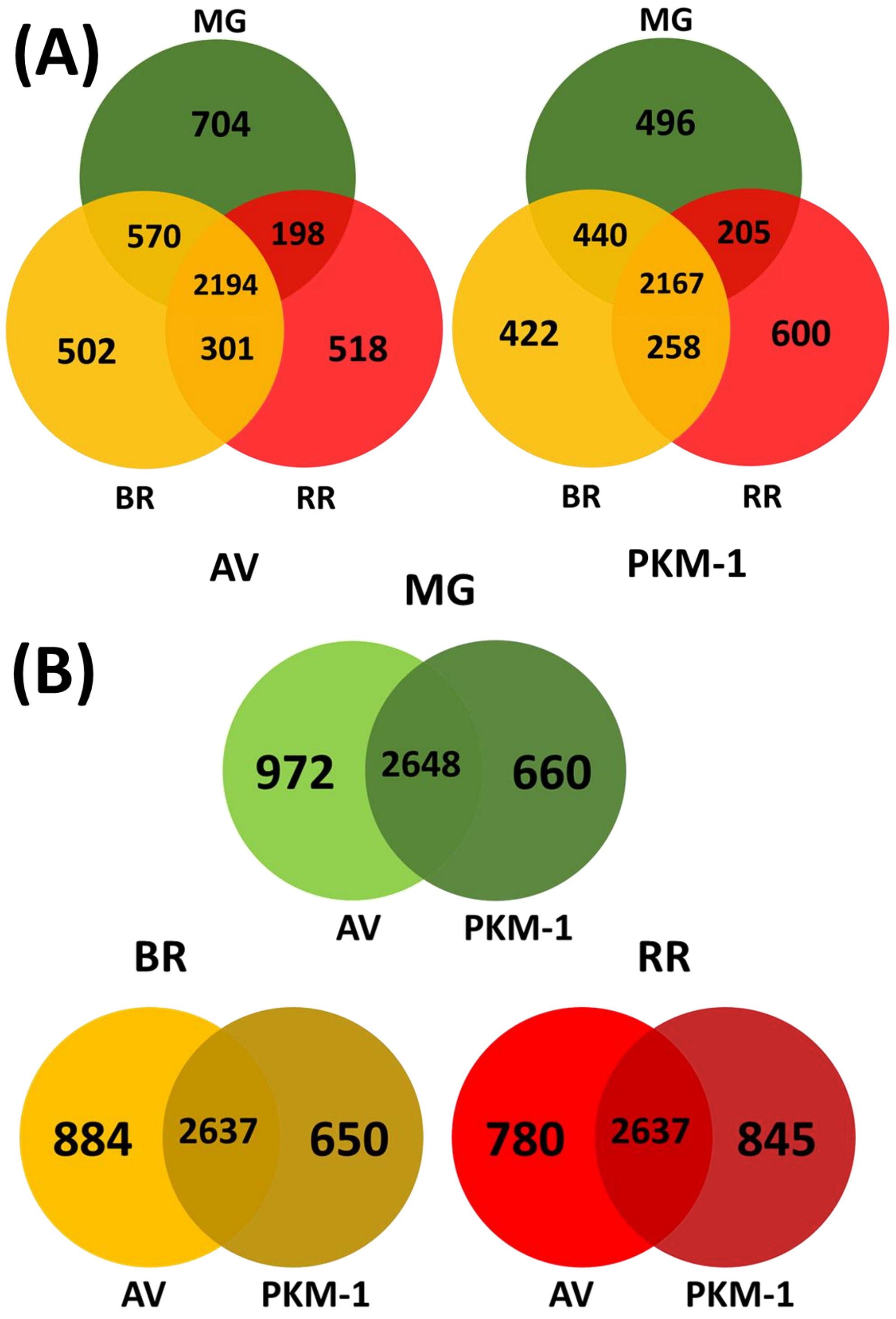


**Figure S5. Venn diagram showing total proteins identified in AV and PKM-1. (A)** Common and unique proteins in AV and PKM-1 at MG, BR, and RR stages of fruit ripening. **(B)** Common and unique proteins between AV and PKM-1 at MG, BR, and RR stage of fruit ripening. The proteins were identified using Sorcerer software version 1 with a minimum of a single peptide match per protein and FDR of 1% (protein level) and 0.1% (peptide level). (For details, **see Dataset 3)**.

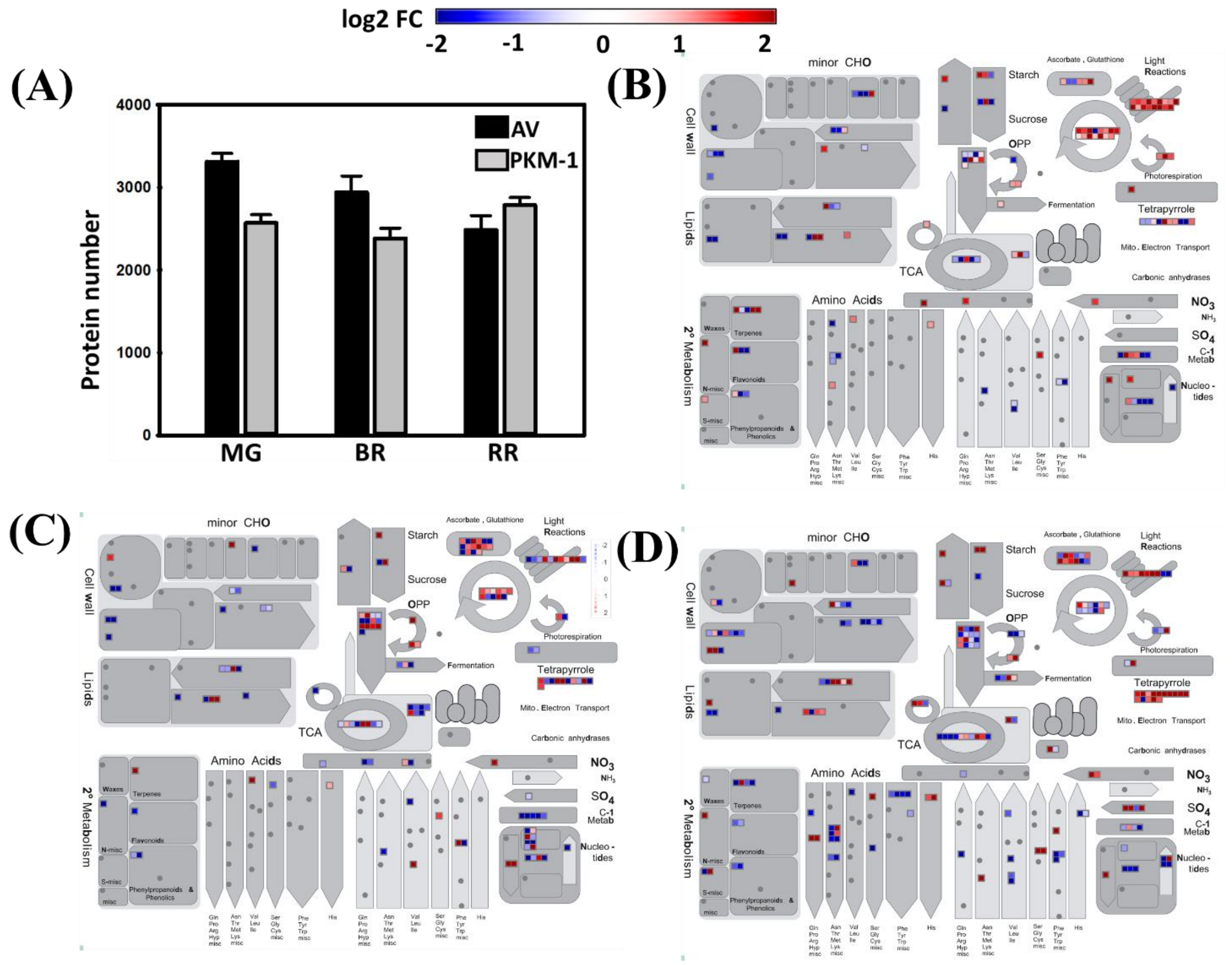


**Figure S6. Total protein identified in AV and PKM-1 and Mapman representations of proteome changes in PKM-1 fruits.** (A) Proteins were identified in AV and PKM-1 at MG BR and RR stage through Sorcerer software version 1. (B-D) Mapman overview of metabolic processes affected in PKM-1 at different stages of fruit ripening. B-D shows processes affected by differentially expressed proteins at MG (B), BR (C), and RR (D) stages. Each square represents one protein. The red square indicates the upregulation of transcripts/proteins, and the blue square indicates the downregulation of transcripts/ proteins. Only significantly different proteins (log2 fold ≥±0.58 , P ≤0.05) are depicted in heatmaps . Data are means ± SE (n = 3 ), P ≤ 0.05 . (For details, **see Dataset 4)**.

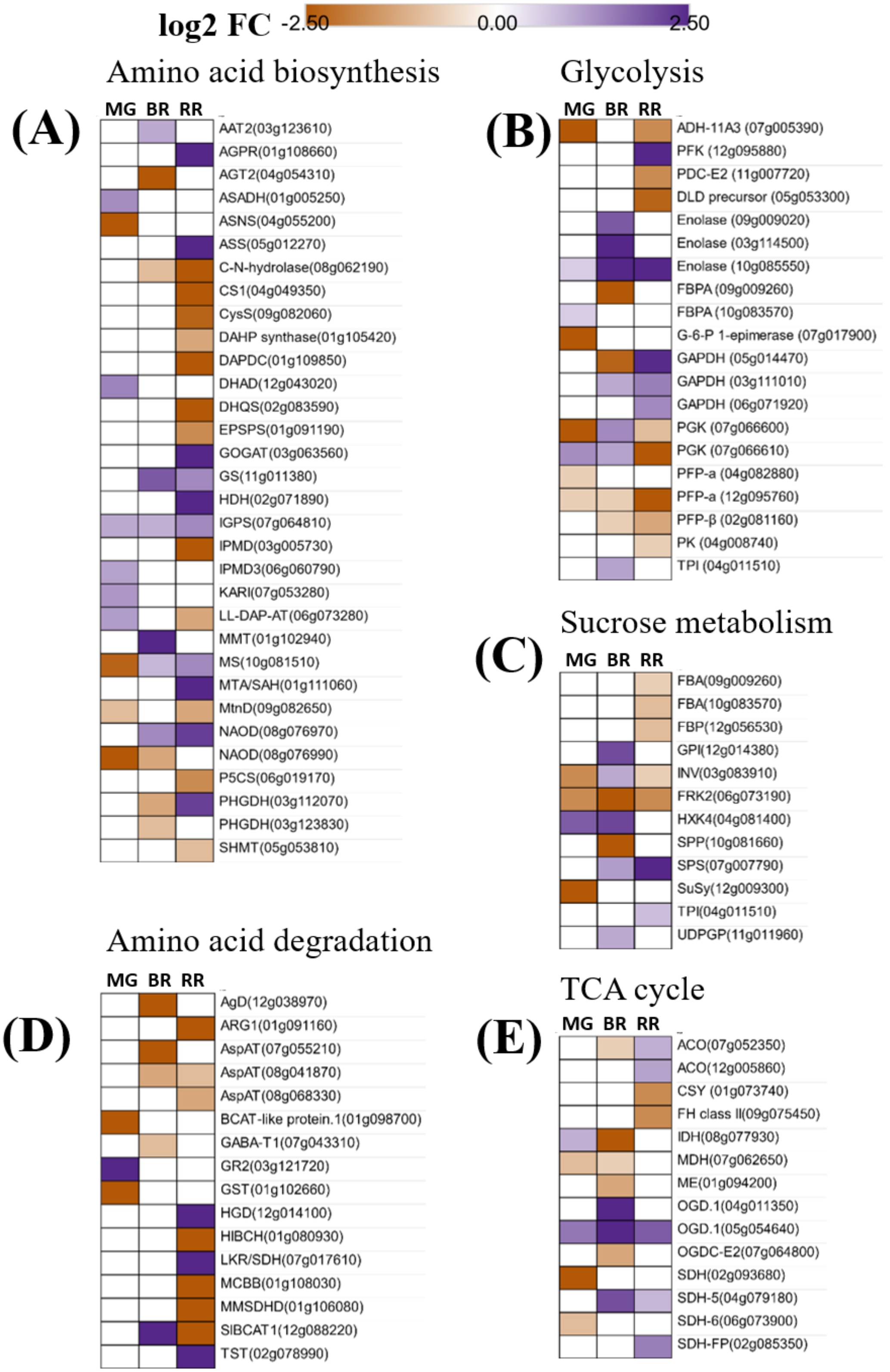


**Figure S7.** Heatmaps representation of differentially expressed proteins affecting metabolic pathways in PKM-1 during fruit ripening. The altered proteins are shown for **(A)** amino acid biosynthesis, **(B)** glycolysis, **(C)** sucrose metabolism, **(D)** amino acid degradation, and **(E)** TCA cycle. Only significantly different proteins (log2 fold ≥±0.58, P ≤ 0.05) are shown in heatmaps. The SOLYC prefix before each protein number is removed for convenience. Data are means ± SE (n = 3), P ≤ 0. 05. See **Dataset 4** for detailed proteome data and the abbreviations of the proteins.

**Figure S8.** The tomato metabolic pathway marked with deleterious genes predicted by SIFT in PKM-1. The genes are highlighted in yellow color on the pathway. (For details, see **Dataset 5**).

Authors: Bo Xue, Pascal Schlapfer, Seung Yon Rhee, Peifen Zhang, Angela Xu, Charles Hawkins, Emily Fryer

# TomatoCyc: *Solanum lycopersicum* Cellular Overview

An online version of this diagram is available at BioCyc.org. Biosynthetic pathways are positioned in the left of the cytoplasm, degradative pathways on the right, and reactions not assigned to any pathway are in the far right of the cytoplasm. Transporters and membrane proteins are shown on the membrane. Periplasmic (where appropriate) and extracellular reactions and proteins may also be shown. Pathways are colored according to their cellular function. Connections between pathways are omitted for legibility.

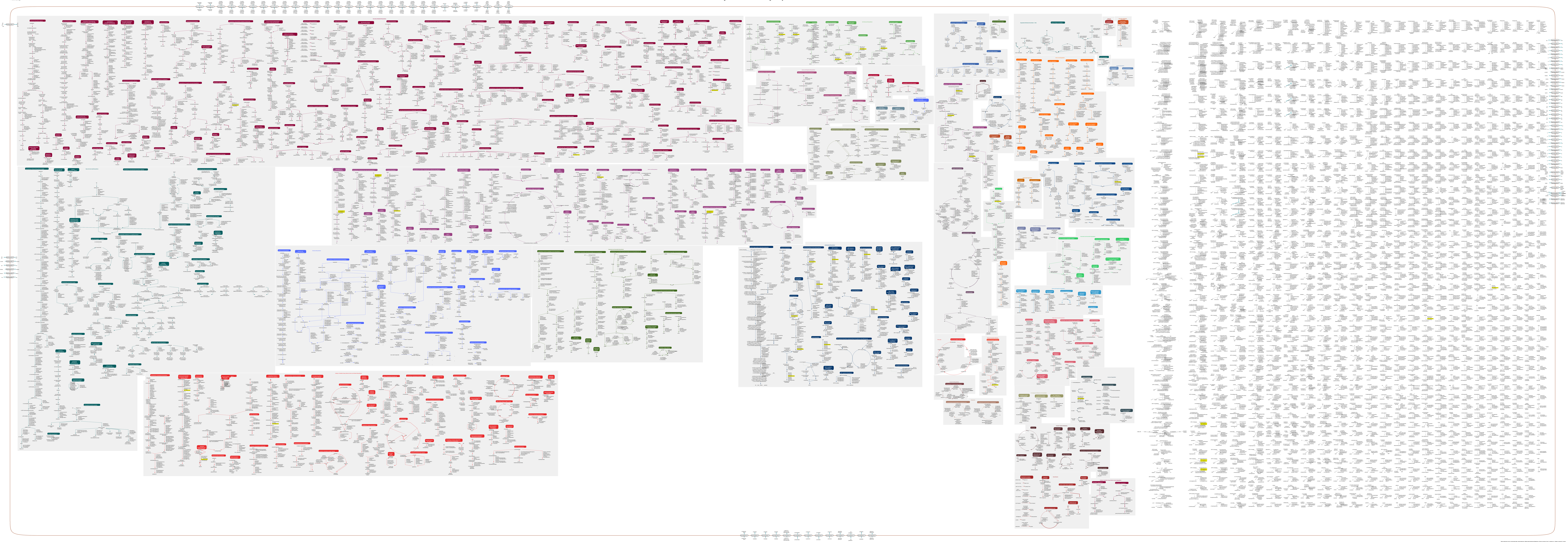

This diagram was automatically generated by SRI International Pathway Tools version 24.0, authors S. Paley and P.D. Karp.
