# Supplementary material for: Reduced γ-glutamyl hydrolase activity likely contributes to high folate levels in Periyakulam-1 tomato
Source: Hortic Res. 2022 Nov 19;10(1):uhac235. doi: 10.1093/hr/uhac235 (PMC9832877; doi:10.1093/hr/uhac235)
Supplement: Web_Material_uhac235 [file web_material_uhac235.zip › Tables S1-4.pdf]

Table S1. Carotenoids profiles of AV and PKM-1 at MG, BR, and RR fruits. Values in bold show the significant difference ($P \leq 0.05$) with respect to AV. Data are mean ± SE (n =4) and expressed as µg/gm FW. The ND (Not detected) refers to the stages where the carotenoid analysis was carried out, but respective carotenoid was not detected, perhaps due to being below the limit of the detection.

**Carotenoid**	**MG**		**BR**		**RR**	
	**AV**	**PKM-1**	**AV**	**PKM-1**	**AV**	**PKM-1**
Lycopene	0.3±0.02	0±0	0.42±0.04	0.58±0.08	56.63±4.56	**78.7±3.05**
β-Carotene	0.68±0.09	0.87±0.21	0.99±0.23	1.56±0.08	2.95±0.21	**4.94±0.2**
Lutein	0.94±0.06	0.99±0.05	0.6±0.05	**2.61±0.14**	0.94±0.12	**2.29±0.22**
Phytoene	ND	ND	ND	ND	32.33±2.55	**50.43±5.31**
Violaxanthin	0.54±0.04	0.5±0.1	0.44±0.07	**0.75±0.03**	ND	ND
Neoxanthin	0.26±0.02	0.96±0.33	0.06±0.01	**0.9±0.05**	ND	ND
β-Cryptoxanthin	ND	ND	ND	ND	2.33±0.49	2.38±0.29
α-Carotene	ND	ND	ND	ND	1.02±0.08	1.18±0.12
ζ-Carotene	ND	ND	ND	ND	8.98±0.92	11.8±1.57
δ-Carotene	ND	ND	ND	ND	2.15±0.47	**3.69±0.38**
γ-Carotene	ND	ND	ND	ND	ND	1.71±0.14

Table S2. The relative proportions of different functional categories of mutations in genes and in noncoding regions including promoters and introns in PKM-1.

Type	SNPs	Genes
Whole Genome	350316	2359
CDS	5664	1765
3' UTR	1323	515
5' UTR	967	430
Nonsynonymous	3568	1377
Synonymous	1921	843
Noncoding	2290	878
START-lost	14	14
STOP-gain	142	118
STOP loss	19-	19
SIFT Deleterious	1307	679

The total number of unique SNPs (350,316) in whole genome of PKM-1 was calculated after subtracting the SNPs present in Arka Vikas compared to reference genome of tomato cultivar Heinz. The total number of 828,076,956 nucleotide predicted by ITAG3.2 tomato genome assembly were taken as denominator to calculate the percent difference of genome of PKM-1 compared to Arka Vikas (350316/828076956 X 100 = 0.0423%).

Table S3. List of primers used for qRT-PCR analysis for folate biosynthesis pathway in AV and PKM-1 fruit. The primers were designed using SOL ITAG 2.3.

′Gene	SGN id.	Primer Sequence (5′→3′)		Amplicon size (bp)
ADCS	Solyc04g049360	Fp	TGAAAGAAGGGCTCATTATGCT	148
		Rp	CTGGATGTGATAGATTTAAGGTTCC	
ADCL1	Solyc11g071280	Fp	TCGATAGGGAAAGCATAAGACAGA	110
		Rp	ATAGTTGAAAATCACCAGGTCCTG	
GCH1	Solyc06g083230	Fp	AGGATGCTGTTAGAGTCCTATTGC	103
		Rp	TGTTCCTTGTCTTAGAGCCTTAGC	
DPP	Solyc03g043860	Fp	ATGAGGAAAATCTTGCATCACACT	116
		Rp	CATACCATTCCCATCCATCACATT	
DHNA	Solyc10g079830	Fp	CAAGTATCCAGAGGTATCTGCTGTT	99
		Rp	GTATCTAATGATCTCGACACCCAAG	
DHFS	Solyc06g051900	Fp	CACTTCGAGTACCCTATGTAGCAAT	113
		Rp	CGTAAAGTTCAGATGCTACATCCTT	
DHFR	Solyc01g109830	Fp	GTTCAGGAAGTTTTGACATTGCTAC	103
		Rp	AATAGAGAGACAATAAGGCGAGGAT	
HPPK-DHPS	Solyc05g012090	Fp	ACTTTATGCAACTTAGGATCAGTGG	114
		Rp	GTTGACGTTAGCTTTAGACTTCTGC	
FPGSm	Solyc04g016550	Fp	GAGCT TGGACAAACG GTAGTATTTA	114
		Rp	TTTCCAGGAGTGTATGAGGGTATAG	
FPGSp	Solyc05g052920	Fp	TACTTCTCTGCTC TACGCTTCAAAT	111
		Rp	AGTTGGCTTTATCTCATCCTTACCT	
GGH1	Solyc07g062270	Fp	GAAGATTTCCCCCCGTGTTGCTAAAGA	128
		Rp	CAATACCCTGAAAAAGCTACTCAAA	
GGH2	Solyc10g007410	Fp	CCCGATTATATATACAGAGCCTCCT	140
		Rp	CTTCAAAGTAGAGACCCTTCTTGCT	
GGH3	Solyc07g062280	Fp	ACCAGAAGCTCAAAAAGTACTTGAC	104
		Rp	GACTGTTGAAAACGTAAACCTCATC	
β-ACTIN	FJ532351.1	Fp	GTCCCTATTTACGAGGGTTATGC	108
		Rp	CAGTTAAATCACGACCAGCAAGATT	
UBIQUITIN 3	X58253.1	Fp	GCCGACTACAACATCCAGAAGG	110
		Rp	TGCAACACAGCGAGCTTAACC	

ADCS, aminodeoxychorismate synthase; *ADCL*, aminodeoxychorismate lyase; *GCHI*, GTP cyclohydrolase I; *DPP*, dihydroneopterin triphosphate pyrophosphatase; *DHNA*, dihydroneopterin aldolase; *DHFS*, dihydrofolate synthetase; *DHFR*, dihydrofolate reductase; *HPPK-DHPS*, hydroxymethyldihydropterin pyrophosphokinase – dihydropteroate synthase; *FPGSm*, folylpolyglutamate synthase (mitochondrial); *FPGSp*, folylpolyglutamate synthase (plastidial); *GGH*, γ-glutamyl hydrolase.

Table S4. List of primers used for qRT-PCR analysis for carotenoid biosynthesis pathway in AV and PKM-1 fruit. The primers were designed using SOL ITAG 2.3

Gene	SGN id.	Primer Sequence (5′→3′)		Amplicon size (bp)
DXS	Solyc01g067890	FP	AAATGGGATCGGTGTAGAGC	115
		RP	TGCTGAGCCATATCCCAATA	
GGPPS2	Solyc04g079960	FP	ATCAATGGAGCAGCTTTGTG	128
		RP	GCGGTTGATAAAACGACGTA	
PSY1	Solyc03g031860	FP	TGAATTAGCACAGGCAGGTC	140
		RP	TCAATTCTGTCACGCCTTTC	
PDS	Solyc03g123760	FP	TATCATCAACGTTCCGTGCT	122
		RP	TATCGGTTTGTGACCAGCAT	
ZISO	Solyc12g098710	FP	AGAGCGTGCTTTTCGTGTATTG	107
		RP	ATTGCCATAACTGCACTCCATC	
ZDS	Solyc01g097810	FP	TCCAAAAGGGCTATTTCCAC	115
		RP	TTGATCCAAGAGCTCCACAG	
CRTISO	Solyc10g081650	FP	GAGATCGCCAAATCCTTAGC	118
		RP	CAGAAAGCTTCACTCCCACA	
LCYB1	Solyc04g040190	FP	CGATGCAACTGGCTTCTCTA	149
		RP	AATGAGAATCTCGCCAATCC	
CYCB	Solyc06g074240	FP	TCTTCTCAAGCCTTTTCCATC	92
		RP	TGGTGGGACTTAGAAAAGAAGG	
LCYE	Solyc12g008980	FP	TTAGTCGCCATTTTCTGCAC	130
		RP	TCACCCTCGCACTCTACAAG	
ZEP	Solyc02g090890	FP	GGTCGTGTTACATTGCTTGG	118
		RP	TGCATGCTTTTTCAAGTTCC	
VDE	Solyc04g050930	FP	GTGCAGCTAATGTTGCCTGT	126
		RP	GGGAGACTGCACACTCATTG	
CYP97A	Solyc04g051190	FP	GTGCCATTGTACCAGCATTG	101
		RP	TGCAGCAACATCAAGCTTTT	
CYPC11	Solyc10g083790	FP	TGCTGAGAGAATGGTGGAGA	107
		RP	GTGCAAGGCCAATAACATCA	
NCED	Solyc07g056570	FP	TGACACCACCAGACTCCATT	130
		RP	ACTTGTTCATCCGGGTTTTC	

DXS, deoxy-xylulose 5-phosphate synthase; *GGPPS*, geranylgeranyl diphosphate synthase; *PSY1*, phytoene synthase 1; *PDS*, phytoene desaturase; *ZISO*, ζ-carotene isomerase; *CRTISO*, carotenoid isomerase; *ZDS*, ζ-carotene desaturase; *LCYB1*, lycopene β-cyclase1; *CYCB*, chromoplast specific lycopene β-cyclase; *LCYE*, lycopene ε-cyclase; *ZEP*, zeaxanthin epoxidase; *VDE*, violoxanthin deepoxidase; *CYP97a*, cytochrome P450 carotenoid β-hydroxylase A29; *CYP97C11*, cytochrome P450 carotenoid ε-hydroxylase C11; NCED, 9-cis-epoxycarotenoid dioxygenase.
